# Real-time fluorescence imaging with 20 nm axial resolution

**DOI:** 10.1038/ncomms9307

**Published:** 2015-09-22

**Authors:** Daniel R. Stabley, Thomas Oh, Sanford M. Simon, Alexa L. Mattheyses, Khalid Salaita

**Affiliations:** 1 Department of Chemistry, Emory University, Atlanta, Georgia 30322, USA; 2 Laboratory of Cellular Biophysics, The Rockefeller University, New York, New York 10065, USA; 3 Department of Cell Biology, Emory University School of Medicine, Atlanta, Georgia 30322, USA

## Abstract

Measuring the nanoscale organization of protein structures near the plasma membrane of live cells is challenging, especially when the structure is dynamic. Here we present the development of a two-wavelength total internal reflection fluorescence method capable of real-time imaging of cellular structure height with nanometre resolution. The method employs a protein of interest tagged with two different fluorophores and imaged to obtain the ratio of emission in the two channels. We use this approach to visualize the nanoscale organization of microtubules and endocytosis of the epidermal growth factor receptor.

## Abstract (web summary)

Mapping the nanoscale height and dynamics of structures within the cell is difficult. Here the authors present a two-wavelength total internal reflection fluorescence method to perform real-time imaging with nanometre axial resolution using a conventional microscope.

## Graphical abstract

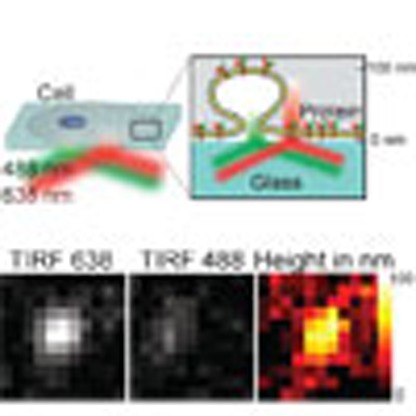

The majority of biological processes involve the intricate assembly of multiple proteins and biomolecules to form nanoscale structures with distinct functions. Accordingly, the advent of super-resolution imaging^1^,^2^,^3^,^4^ has greatly advanced our understanding of a range of biological structures from focal adhesions^5^ to the neuronal cytoskeleton^6^. Although recent developments in super-resolution imaging have been revolutionary in improving optical resolution, they are limited in resolving real-time dynamics along the *z*-direction, perpendicular to the sample.

One of the simplest methods to improve axial resolution is total internal reflection fluorescence (TIRF) microscopy^7^,^8^. TIRF takes advantage of an evanescent excitation field that decays exponentially from the coverslip, limiting the illumination depth to ∼100–200 nm. Given the exponential decay of the evanescent field, a number of TIRF-based axial imaging approaches have been developed by simply recording changes in intensity^9^, collecting a sequence of images at different incidence angles^10^ or normalizing the TIRF image to a widefield image^11^. Among these methods, differential interference nanometry (DiNa) is robust to changes in protein clustering, as axial imaging is achieved by using the ratio between widefield fluorescence and TIRF. This approach is advantageous when studying processes involving protein oligomerization and trafficking. However, DiNa reports the difference in height between two different proteins that need to be matched in expression level. Moreover, DiNa and other TIRF-based approaches are hampered by the requirement of capturing multiple images to obtain the axial *z*-position and thus limits temporal resolution. This is particularly problematic when imaging the dynamics of most biological structures at the plasma membrane. Accordingly, it is not possible to distinguish rapid changes in protein density from dynamic movements in *z*-position. For example, both the protein composition and the axial position of a clathrin-coated pit can rapidly (∼ms–s) change during pit maturation, and consequently direct evidence of this process is lacking. Similarly, the non-TIRF-based technique scanning angle interference microscopy provides ∼10 nm axial resolution, but requires collecting a sequence of ∼10 images at different incidence angles and is thus appropriate only for relatively static structures^12^. Therefore, existing techniques are suitable for imaging relatively static structures, such as mature focal adhesions and stationary microtubules, but not appropriate to the study of dynamic protein complexes. Thus, there is a significant need for methods to acquire the nanometre position of protein structures in real-time.

Herein we present a technique, simultaneous two-wavelength axial ratiometry (STAR), for measuring the height of tagged molecules with nanometre resolution in real-time.

## Results

**Figure 1:**
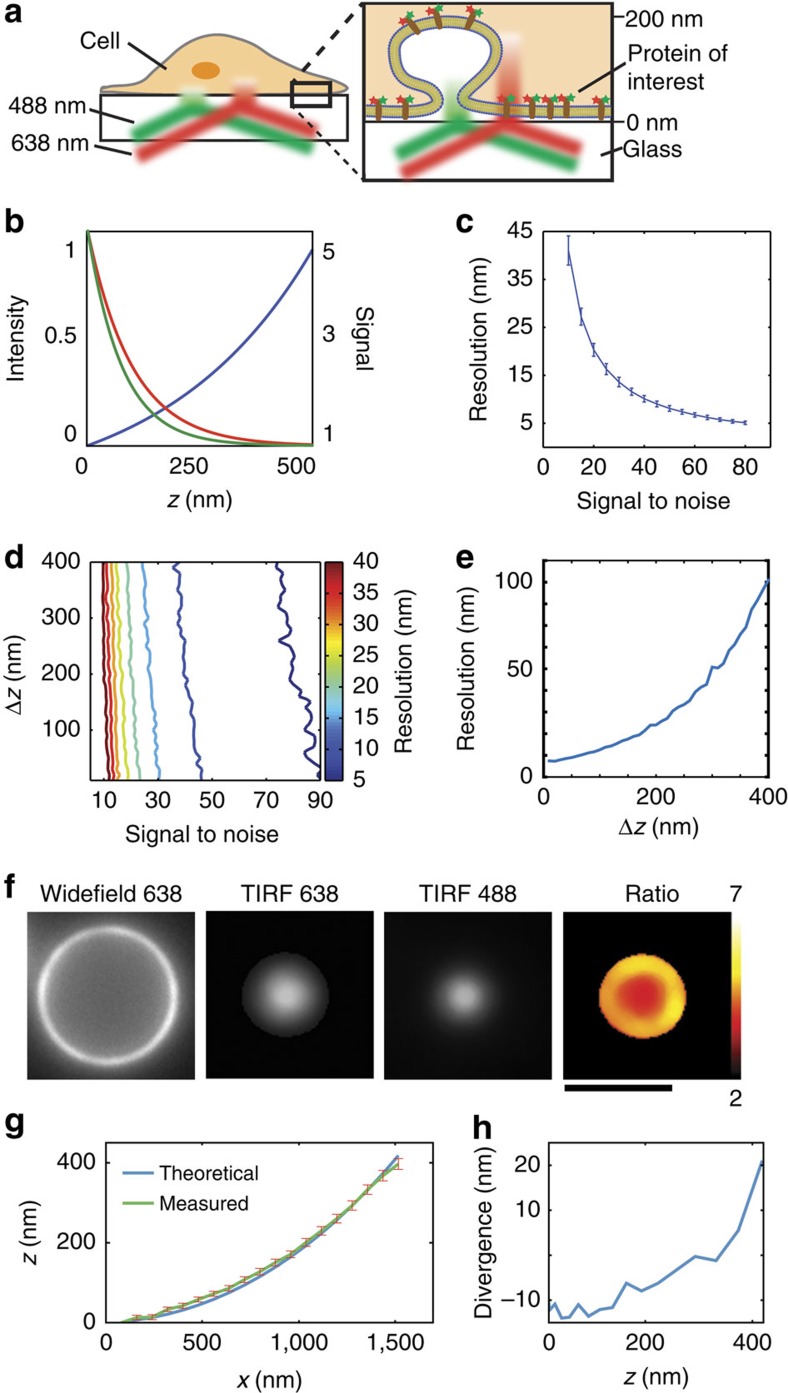
STAR theory and demonstration. (**a**) Schematic showing the basic principal of STAR: the *z*-position of a protein labelled with two spectrally distinct fluorophores is determined by imaging in two TIRF channels and calculating the intensity ratio. (**b**) The theoretical excitation profile of the evanescent field generated by 488 nm (green) and 638 nm (red) excitation and the ratio of 638/488 as a function of distance from the coverslip (*z*) (**c**) Theoretically calculated resolution as a function of S/N ratio; error bars are s.d. for *z* from 5 to 400 nm. (**d**) Isosurface plot of STAR resolution as a function of S/N ratio and *z*-position. Each line represents a different resolution, from 5 nm (blue) to 40 nm (dark red). The simulated conditions were: *λ*_1_=638; *λ*_2_=488; *n*_1_=1.515; *n*_2_=1.33; *θ*=70°. (**e**) Theoretical resolution as a function of *Δz* for an object with S/N=70 at *z*=0. (**f**) Representative images of a dual-tagged microsphere. Scale bar, 5 μm. (**g**) The theoretical (blue) and measured (green) *z*-position as a function of distance from the centre of the bead (*x*). The measured plot is an average of 19 beads of identical diameter. (**h**) Divergence between the average measured and theoretical *z*-position as a function of *z*.

In STAR, the sample is excited with two wavelengths, each with a unique evanescent decay constant, to measure the axial position of dual-tagged molecules (Fig. 1a). The profile of the evanescent field is defined as:


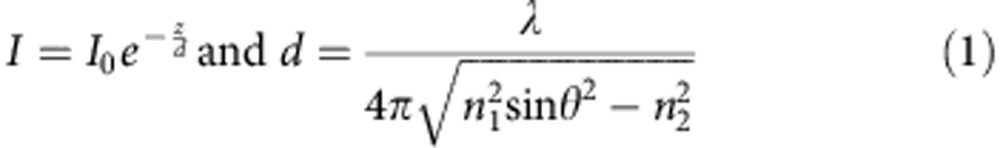


where *I*_0_ is the intensity at *z*=0, *n*_1_ and *n*_2_ are the indices of refraction of the glass coverslip and the sample, respectively, *λ* is the excitation wavelength and *θ* is the angle of incidence. The intensity profile of the excitation field is unique for each wavelength, resulting in a ratio of fluorescence emission intensity between the two channels that is dependent on the nanometre axial position (Fig. 1b). Although the absolute intensity in each channel is proportional to the number of molecules, the ratio of the two channels is independent of molecular density and depends only on the *z*-position. Therefore, the relative *z*-position can be calculated by:


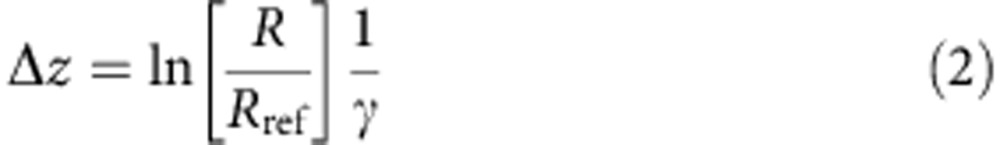


where *R* is the ratio of fluorescence intensity between two channels (*R*=*I*^*λ*2^*/I*^*λ*1^), *R*_ref_ is the ratio of fluorescence intensities at the reference position (*R*_ref_=*I*^*λ*2^_ref_*/I*^*λ*1^_ref_) and *γ* is a constant derived from the evanescent fields (see Methods). In our experiments, *λ*_1_=488 nm and *λ*_2_=638 nm, commonly used wavelengths for cellular imaging (Fig. 1a). Thus, in principle, the nanometre height of assembling or disassembling structures can be measured in real-time from the ratio of fluorescence intensities at these two wavelengths.

To determine the theoretical resolution of STAR, we simulated raw fluorescence intensity data incorporating photon (shot) noise, while also varying protein concentration, *z*-position and incidence angle. This analysis revealed that the theoretical resolution depends heavily on the signal-to-noise ratio (S/N) and can be as small as 5 nm when an object is bright (Fig. 1c). Importantly, resolution is primarily dependent on emitter brightness (that is, S/N ratio), regardless of the *z*-position of the object of interest (Fig. 1d). Although Fig. 1d may seem counterintuitive, this relationship assumes a constant S/N ratio at different *z*-positions, which does require increasing the number of emitters to offset the decay in evanescent field intensity. If, however, the number of fluorophores remains constant as the object moves away from the coverslip, then both the S/N ratio and resolution of STAR decrease. This situation is plotted in Fig. 1e, assuming an initial S/N ratio of 70 near the coverslip. For example, the *z*-resolution of a structure near the coverslip is predicted at 8 nm and this increases to 43 nm when the object translocates 300 nm away from the coverslip. This change in resolution is due to a decrease in S/N ratio, as the object moves through the evanescent field, away from the coverslip. Interestingly, the resolution can be tailored to better accommodate specific *z*-positions by varying the incidence angle (Supplementary Fig. 1).

To validate the accuracy of STAR, we imaged fluorescently labelled silica microspheres (beads) with a low refractive index compatible with TIRF^13^. When imaged using an inverted microscope, the height of the bead (*z*) increases with distance from the bead centre (*x*) and can be described by simple trigonometry (Supplementary Fig. 2). The beads were functionalized with biotin and then modified with streptavidin dual-labelled with Alexa 647 and Alexa 488. Labelled microspheres were mounted on coverslips in refractive index-matched media and imaged using STAR. To determine the labelling uniformity and diameter of individual beads, conventional TIRF and widefield images were acquired and analysed (Fig. 1f). The height profile of uniformly labelled beads was then calculated from the fluorescence intensity ratio (Supplementary Note 1 and Supplementary Fig. 3). The experimentally measured average height profile for 19 beads (diameter=6.08 μm) was in agreement with the predicted height profile (Fig. 1g). The deviation of the experimental profile from the predicted height increased at greater *z*-positions, a trend expected given the lower S/N ratio at greater *z* (Fig. 1h). The average divergence of the experiment from theory was <20 nm over a range of *z*-positions from 0 to 400 nm. The lower experimental precision than that predicted from the simulation (compare Fig. 1c–e with Fig. 1h) is probably due to variations in the physical uniformity of the beads as well as our ability to accurately measure their diameters.

**Figure 2:**
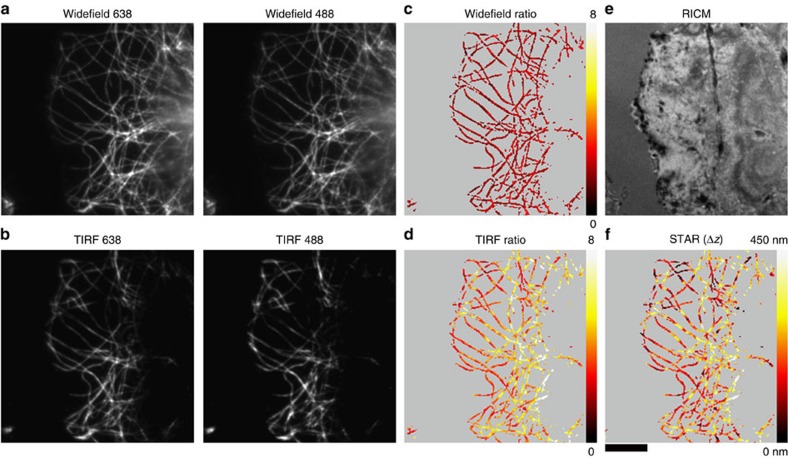
Measuring the height of microtubules labelled with standard immunofluorescence using STAR. Representative images of COS-7 cells stained with biotinylated anti-α-tubulin primary antibody and dual-tagged streptavidin (Alexa 647 and Alexa 488). Cells were imaged with (**a**) widefield and (**b**) TIRF microscopy in the 488- and 638-nm channels. The ratio of the 638/488 channels for (**c**) widefield and (**d**) TIRF. (**e**) RICM images showing the adhesion profile of the cell. (**f**) STAR *Δz* image indicates the nanometre height of microtubules. Scale bar, 10 μm.

Having established the accuracy of the technique, we next used STAR to create a height map of microtubules in fixed cells. COS-7 cells were fixed and microtubules labelled with a biotinylated primary antibody and Alexa 488 and Alexa 647-tagged streptavidin. Cells were imaged with widefield (Fig. 2a) and TIRF (Fig. 2b) in both the 488- and 638-nm channels. Compared with widefield, only microtubules near the coverslip are visible in TIRF. The ratio of the widefield channels was uniform (1.9±0.3; *σ*=s.d. comparing widefield images) for all microtubules in the cell (Fig. 2c). This was expected, because the widefield ratio measures the product of the detection efficiency of the Alexa 488 and Alexa 647 dyes and their respective labelling ratios. We further verified that the density of labelled streptavidin did not affect the ratio by using surfaces functionalized with varying densities of dual-tagged streptavidin (Supplementary Fig. 4). In contrast, the ratio of the TIRF images varied from 1 to 8 along individual microtubules, displaying an overall decrease near the cell edge (Fig. 2d) corresponding to the cell–substrate contact regions captured with reflection interference contrast microscopy (RICM; Fig. 2e).

To determine the nanometre *z*-position, the TIRF images for each channel were background subtracted and divided to obtain a ratio (Supplementary Fig. 5). The signal corresponding to *z*=0 was determined from the intensity ratio of the widefield images. The STAR height map was then calculated using equation (2). RICM shows a tight association between the cell membrane and the substrate near the cell edge; this contact is reduced towards the centre of the cell (Fig. 2e). Likewise, the microtubules near the cell edge are generally located between 0 and 100 nm, and increase in height towards the centre of the cell (Fig. 2f). These values agree with published *z*-positions obtained using scanning-angle fluorescence interference contrast microscopy^12^. One important caveat is that when two structures are located within the same diffraction limited spot but at different heights (microtubules crossing), the STAR measurement is biased. For example, two objects at *z*=50 and 100 nm will appear as a single object at *z*=68 nm, rather than at *z*=75 nm.

To demonstrate the dynamic imaging capability of STAR, we next measured the nanometre *z*-dynamics of the epidermal growth factor receptor (EGFR) during ligand binding, clustering and internalization at the plasma membrane. EGFR endocytosis involves a host of rapidly assembling proteins and plasma membrane deformations, and this process can be triggered by addition of its ligand, EGF^14^. Although the overall cellular response to EGF is well characterized, the assembly dynamics of individual vesicles are difficult to capture.

**Figure 3:**
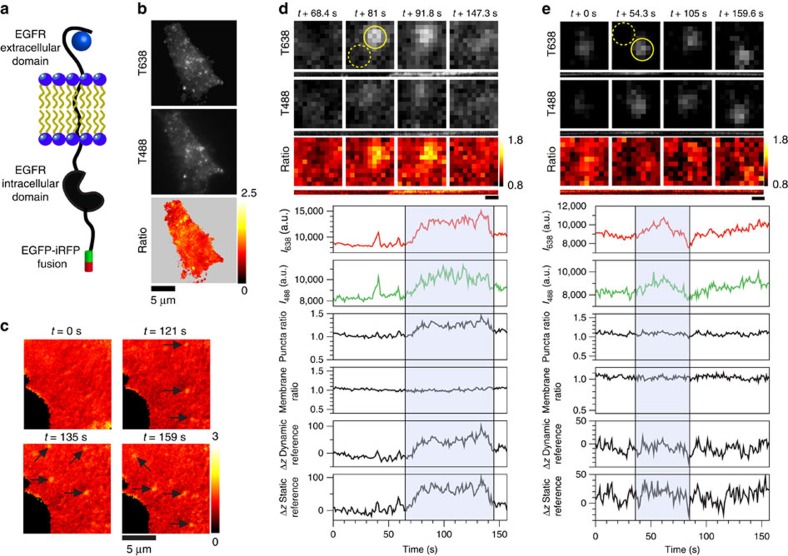
Imaging EGFR endocytosis in real-time with STAR. (**a**) Schematic of EGFR-EGFP-iRFP construct. (**b**) COS-7 cell expressing EGFR-EGFP-iRFP after EGF stimulation imaged in TIRF 638 and TIRF 488, and the 638/488 ratio. (**c**) Four representative 638/488 ratio images from a time-lapse video captured after EGF stimulation. Individual puncta are identified with black arrows. (**d**,**e**) Traces and representative images of individual EGFR puncta. Below each set of fluorescent images is a kymograph of the puncta over the entire time series. Representative ROIs are depicted with solid yellow circles (puncta) and dashed yellow circles (membrane). Graphs, from top to bottom, show the mean intensity of the puncta in the 638 channel and the 488 channel, the ratio of 638/488 for the puncta, the ratio of 638/488 for adjacent membrane, the calculated *Δz* with a dynamic membrane reference over time and the calculated *Δz* using a static reference. Scale bar, 500 nm.

We tandem tagged the carboxy terminus of EGFR with both enhanced green fluorescent protein (EGFP) and infrared fluorescent protein (iRFP)^15^, creating EGFR-EGFP-iRFP (Fig. 3a). These fluorophores were selected to maximize the STAR dynamic range, while also minimizing fluorescence resonance energy transfer (Supplementary Figs 6 and 7, and Supplementary Note 2). Transiently transfected COS-7 cells expressing EGFR-EGFP-iRFP were simultaneously imaged to generate STAR data (Fig. 3b). The signal in both fluorescence channels was generally diffuse and localized to the plasma membrane. Within 5 min of stimulation with 2 nM EGF, dynamic formation and disappearance of EGFR puncta was observed, confirming biological activity of the dual-tagged construct (Fig. 3c). As a control to establish the requirement for the dual-tagged construct, we compared COS-7 cells expressing EGFR-EGFP-iRFP to those expressing two proteins, EGFR-EGFP and EGFR-iRFP. We found that the ratio of the iRFP and EGFP emission intensities fluctuated significantly when the individual tagged proteins were mixed, producing both positive and negative *Δz* measurements; in contrast, the dual-tagged construct displayed a narrow range of ratios. The fluctuations were attributed to differential expression and localization of the individually tagged target proteins (Supplementary Fig. 8).

Following EGF stimulation, the accumulation of EGFR within diffraction limited spots in both channels was used to identify potential endocytic pits for further analysis (Fig. 3c). Images and analysis highlighting the dynamics of a representative EGFR puncta are shown in Fig. 3d. The EGFP and iRFP fluorescence intensities were measured over time from a region of interest (ROI) corresponding to the puncta. The ratio of these values directly provides *R*, as defined in equation (2). Importantly, *R* can be used to obtain an absolute nanometre *Δz* in two ways. One method is a dynamic reference, where the nanometre position of the EGFR pit is determined relative to the surrounding plasma membrane height in each frame (Fig. 3d, ROI denoted as dashed yellow circle and plot of membrane ratio). This type of measurement produces a *Δz* that is robust against undulations in the position of the cell membrane (Fig. 3d, dynamic reference plot). Alternatively, the nanometre *Δz* position of an individual puncta can be referenced to a static position before formation of the pit by averaging the initial 20 frames of the same ROI before EGFR accumulation (Fig. 3d, static reference plot; Supplementary Fig. 9). Comparison of the *Δz* plots obtained using static and dynamic referencing indicates nearly identical results, suggesting minimal membrane fluctuations in the time window of observation. This is likely dependent on cell type, ligand and surface functionalization. It is noteworthy that all *Δz* measurements were corrected for differential photobleaching of the two fluorophores by applying a correction to the time-lapse data (Supplementary Fig. 10).

Based on the analysis of a large data set including 60 EGFR puncta from 3 cells using a static reference, several distinct behaviours falling into 3 categories were identified. Approximately one-third of the observed puncta were in the first category, where an increase in *z* correlated with an increase in fluorescence intensity in both channels. Typically, the fluorescence reached a value that was twofold over background, while *Δz* reached a height of 80 nm that was maintained for tens of seconds. Finally, this was followed by a rapid return to the baseline *z* that coincided with loss of fluorescence and disappearance of the puncta from the evanescent field (Fig. 3d). The measured maximum *z* of 80 nm is consistent with a 100-nm radius sphere (vesicle) imaged using STAR and agrees with the dimensions of clathrin-coated pits determined by cryo-electron microscopy (Supplementary Note 3).^16^ The recorded height dynamics are consistent with two models for internalization wherein EGFR is recruited to preformed pits or EGFR recruitment and pit maturation occur simultaneously. Therefore, the data agree with the current hypothesis wherein cargo is sorted into pre-formed pits and provides additional detail as to the *z*-dynamics at the time of cargo selection^17^,^18^.

In the second category, puncta had an accumulation of EGFR and increasing fluorescence intensity that was not associated with displacement in *z* (Fig. 3e). This probably represents EGFR clustering and oligomerization on the plasma membrane not associated with trafficking in the experimental time frame. The persistence of EGFR clusters without endocytosis suggests that these assemblies may be functioning as a signalling platform. The remaining third of observed puncta did not fall into either of the first two categories and were placed in a third group. The dynamics in this category were heterogeneous, encompassing cases that displayed varying *Δz* and EGFR intensity. Of note are puncta where an increase in EGFR intensity was associated with a decrease in height (possibly an EGFR containing vesicle trafficking towards the plasma membrane). Taken together, these observations indicate that EGFR clustering is highly heterogeneous and does not always represent successful endocytosis.

## Discussion

We have developed a simple turn-key strategy based on a commercial TIRF microscope to determine the *z*-position of proteins within a cell with nanometre resolution in real-time. The microtubule height maps and tracking of EGFR endocytosis demonstrate compatibility with widely available organic dyes and conventional fluorescent proteins. A central advantage of the method is real-time imaging of protein dynamics. This is certainly the case when STAR is compared with scanning angle fluorescence interference contrast microscopy^12^. One caveat for STAR is that the localization is limited to fluorophores excited by the evanescent field. We demonstrate this capability by imaging EGFR endocytosis dynamics, providing direct evidence in support of two of the long-standing models describing the mechanism of clathrin-mediated endocytosis. Although some trajectories are in agreement with existing models, our data highlights alternate dynamics of EGFR that are difficult to measure with existing techniques. Importantly, STAR will likely open the door to gaining new insights into the mechanisms of trafficking and assembly of hundreds of membrane-associated proteins central to many of essential cellular processes in a range of living systems.

## Methods

### Theory

The evanescent field produced by two excitation wavelengths *λ*_1_ and *λ*_2_ are


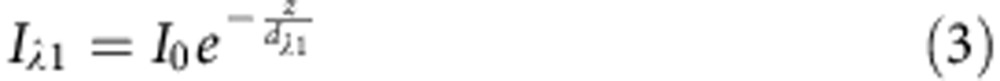



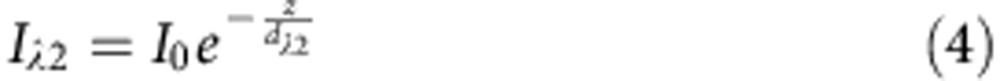


The ratio of intensities, assuming constant *n*_1_, *n*_2_ and *θ* can be written as


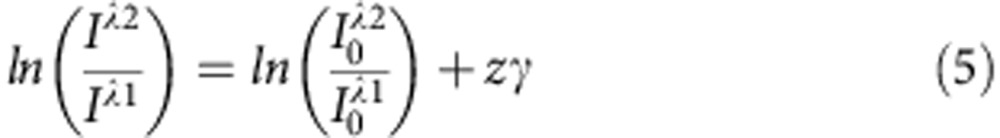


where


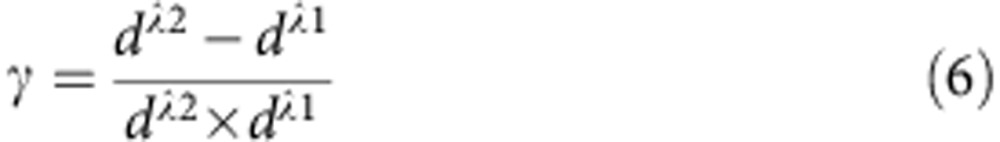


Solving for *z* gives


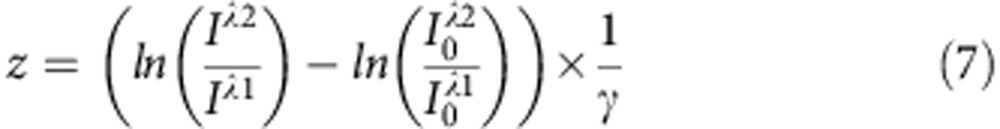


To bypass the requirement of measuring *I*_0_, a relative *z*-position, *Δz*, can be calculated as a ratio of intensity ratios. The reference ratio can be measured in several ways: at *t*=0, in a specific region of the sample, from a widefield image or on a calibration sample known to be restricted in *z*.


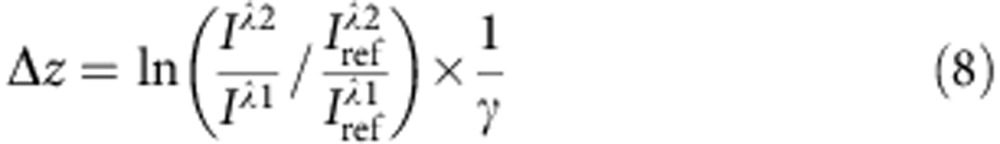


It is worth noting that this is equivalent to equation (2) where *R* is the ratio of fluorescence intensity between *λ*_2_ and *λ*_1_, and *R*_ref_ is the ratio of fluorescence intensities between *λ*_2_ and *λ*_1_ at a reference *z*-position. This can be defined in different ways depending on the experiment. There are two approaches for measuring the reference ratio (*R*_ref_)—static and dynamic. In the static case, *R*_ref_ is measured once and applied to all subsequent data. A static *R*_ref_ can be measured in several ways: at the coverslip sample interface, from an ROI within the plasma membrane at *t*=0, from a ratio of widefield images or from an earlier time point for the object of interest. In contrast, *R*_ref_ can also be determined dynamically. In this case, each time point in a series has its own *R*_ref_ measurement, obtained from an ROI within the same image (for example, dashed yellow circle Fig. 3d). In our analysis of EGFR, we find that the dynamic and static referencing approaches lead to similar results within the time window of the experiment (∼1 h).

### Cell culture

COS-7 cells were cultured in DMEM (Mediatech) supplemented with 10% fetal bovine serum (Mediatech), sodium pyruvate (1 mM, Sigma), L-glutamine (2.1 mM, Mediatech), penicillin G (100 IU ml^−1^, Mediatech) and streptomycin (100 μg ml^−1^, Mediatech), and were incubated at 37 °C with 5% CO_2_. Cells were passaged at 80%–90% confluency and plated at a density of 25% using standard cell culture procedures.

### Microscope setup

Images were acquired on a Nikon Eclipse Ti TIRF microscope with Elements software (Nikon). The microscope is equipped with an Evolve electron-multiplying CCD (charge-coupled device; Photometrics) used for cell imaging and a CoolSnap CCD camera (Photometrics) used for bead imaging, an Intensilight epifluorescence source (Nikon), a CFI Apo × 100 1.49 numerical aperture objective, and 488 nm (10 mW) and 638 nm (20 mW) laser lines. The microscope was equipped with a Quad Band (405/488/561/638m) laser TIRF filter cube with emission bands 500/25 and 730/55, and a cube for RICM (Chroma). EGFR internalization was imaged with an optical splitter, the Opto-Split III (Cairn Research), using 500/25 and 730/55 emission filters and a 600lp dichroic to split the fluorescence emission onto separate regions of the camera.

### Plasmids

We used EGFR (Rat) tagged with EGFP (pEGFP-N1-EGFR)^19^. EGFR tagged with EGFP and iRFP (EGFR-EGFP-iRFP) was constructed as follows: first, iRFP was cloned out of Addgene plasmid 31856 (pShuttle CMV iRFP) using PCR. (This version of iRFP is now known as iRFP 713.) The PCR product was ligated into the AgeI site in the EGFP-EGFR plasmid creating the EGFR-EGFP-iRFP construct. EGFR tagged with iRFP alone was constructed by ligating iRFP into pEGFR-N1-EGFP in the place of EGFP. Cloning was performed by Oskar Laur at the Emory University cloning core.

### Silica microsphere labelling and imaging

Five-micrometre aminated silica microspheres (Bang's Labs) were functionalized with N-hydroxysuccinimide ester (NHS) biotin (Pierce), following the manufacturer's protocols. Biotinylated beads were purified via centrifugation and resuspended in 1 × PBS (3 cycles, 10 min per cycle). The beads were then incubated with dual-tagged streptavidin (prepared by standard reaction with succinimidyl esters of Alexa 488 and Alexa 647 (Life Technologies) for 20 min and re-purified. For imaging, beads were added to wells of a glass-bottomed 96-well plate (Nunco) in 63.5% glycerol formulated to match the index of refraction of the beads (*n*=1.43). Beads were imaged with sequential TIRF 638, TIRF 488, widefield 638 and widefield 488 excitation.

### Microtubule imaging

COS-7 cells were seeded into wells of a glass-bottomed 96-well plate (Nunco) and grown overnight. The next day, cells were rinsed with 5 ml 1 × PBS and incubated with 4% paraformaldehyde (in 1 × PBS) for 12 min. Cells were then rinsed with 5 ml 1 × PBS and permeabilized for 5 min with 0.1% Triton-X in 1 × PBS. Cells were then rinsed with 5 ml PBS followed by incubation with 1% BSA in 1 × PBS for 1 h at room temperature. After blocking, cells were labelled with a biotinylated α-tubulin primary antibody (Life Technologies) at 1:400 dilution in 1% BSA (in 1 × PBS) for 1 h, followed by rinsing and incubation with 1 μg dual-tagged streptavidin in 200 μl 1% BSA in 1 × PBS. Following fixation and staining, cells were imaged with the quad-band TIRF cube and alternating TIRF 488 and TIRF 638 excitation.

### EGFR imaging

COS-7 cells were grown overnight on glass coverslips and subsequently transfected with Lipofectamine 2000, following the manufacturer's protocols. The cells were incubated in serum-free media for 1 h before imaging. In the case of cells transfected with iRFP constructs, the serum-free media incubation included 5 μM biliverdin (the co-factor for iRFP). Time-lapse imaging was performed on individual cells following addition of 2 nM EGF (R&D Biosystems). Five-minute movies were acquired with 200 ms exposure times and a 300-ms delay between frames. All live cell data were acquired using the Cairn OptosplitIII setup described above.

### Theoretical analysis

The effect of noise on STAR measurements was evaluated with theoretical modelling. A virtual STAR experiment was constructed in Matlab with the starting conditions chosen to closely match experimental conditions: *λ*_1_=488 nm; *λ*_2_=638; *n*_1_=1.515; *n*_2_=1.33; *θ*=70°. All analysis was conducted on a pixel-by-pixel basis. *I*_488_ was defined by a given intensity at *z*, all other intensities, *I*_488_ and *I*_638_ at *z*=0 and *I*_638_ at *z* were calculated from the evanescent field equations assuming *I*_488_=*I*_638_ at *z*=0. In Fig. 1e, S/N ratio was defined at *z*=0 for both 488 and 638, and the intensity at all other *z*-positions was generated from the evanescent field profile. Measurements were simulated 1,000 × with Poisson-distributed shot noise. Distance from the coverslip, *z*, was calculated (equation (2)). Resolution was defined as the s.d. of the residual of the calculated *z*-position from the true position. This was repeated for S/N values ranging from 10 to 80 and *z*-positions from 5 to 400 nm.

## Additional information

**How to cite this article:** Stabley, D. R. *et al*. Real-time fluorescence imaging with 20 nm axial resolution. *Nat. Commun.* 6:8307 doi: 10.1038/ncomms9307 (2015).

## Supplementary Material

Supplementary Information Supplementary Figures 1-10 and Supplementary Notes 1-3
