# Supplementary material for: Real-time fluorescence imaging with 20 nm axial resolution
Source: Nat Commun. 2015 Sep 22;6:8307. doi: 10.1038/ncomms9307 (PMC4595625; doi:10.1038/ncomms9307)
Supplement: Supplementary Information — Supplementary Figures 1-10 and Supplementary Notes 1-3 [file ncomms9307-s1.pdf]

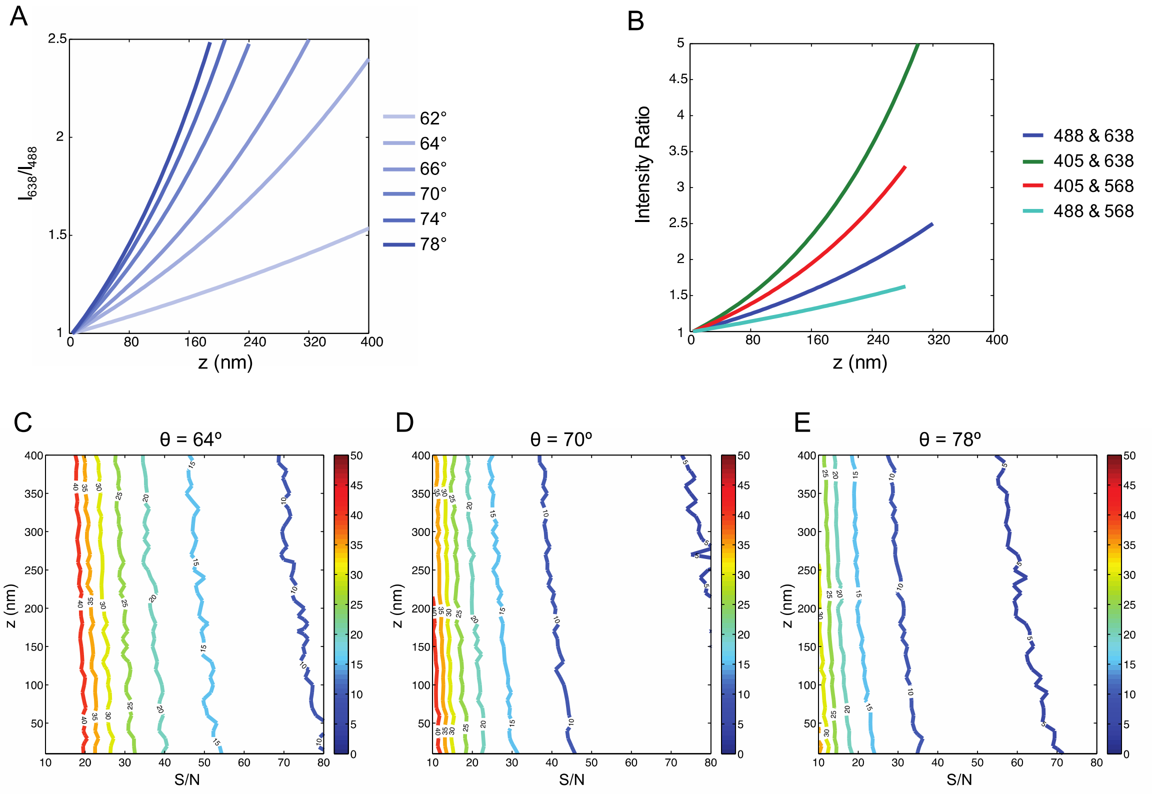

Supplementary Figure 1: Theoretical modeling of STAR A) The ratio of the 638 and 488 nm TIRF excitation fields as a function of distance from the coverslip/sample interface for incidence angles ranging from θ = 62°-78°. The data is plotted to a z where I_{638} = 5% I_{638} at z = 0. The incidence angle can be used to control the rate of change of the I_{638} / I_{488} ratio as well as the z depth to which the technique is applicable. B) The ratio of excitation fields for different wavelength combinations at where θ = 66° C-E) Isosurface contour plots of the theoretical z resolution as a function of the S/N of the 488 nm channel. Parameters were wavelengths 638 and 488 and an incidence angle of C) θ = 64° D) θ = 70° E) θ = 78°. For all calculations, n_1 = 1.515 and n_2 = 1.33.

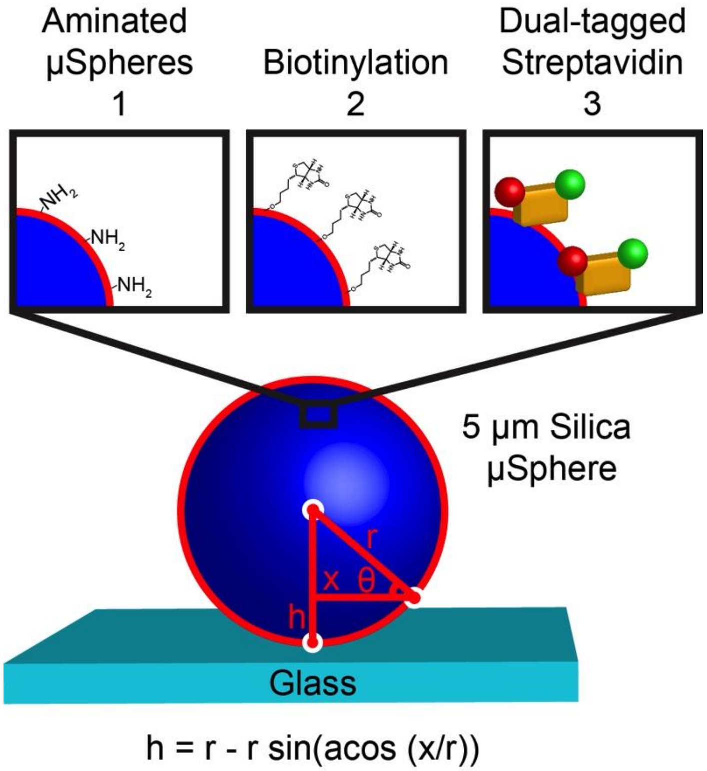

Supplementary Figure 2: Determining the accuracy of STAR Schematic showing the silica µSpheres (beads) used for this experiment; the relevant trigonometry and equations are included. The biotin-streptavidin labeling strategy is illustrated.

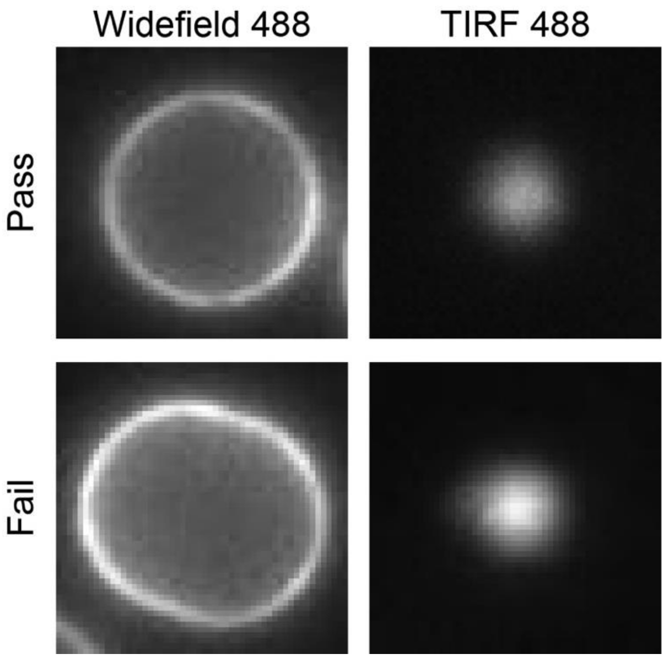

Supplementary Figure 3 Representative images of beads that were analyzed (pass) or discarded (fail).

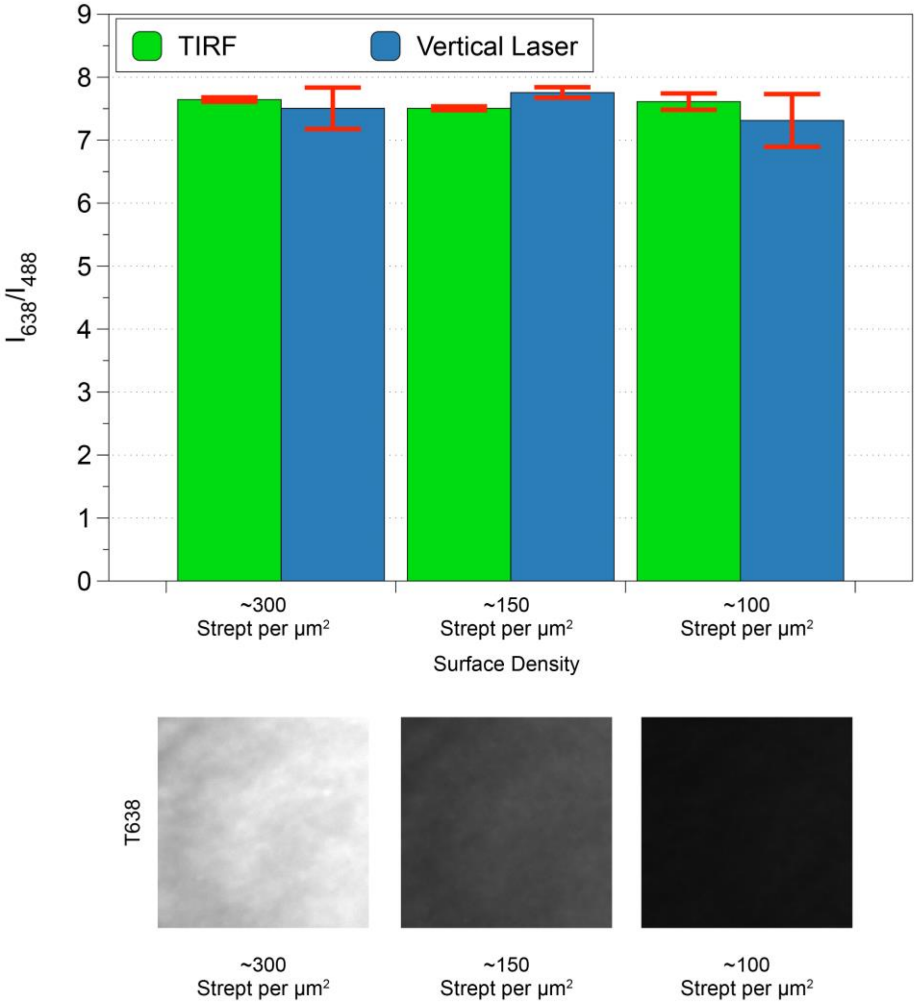

Supplementary Figure 4: I_{638} / I_{488} does not vary with labeling density. Supported lipid bilayers were labeled with Alexa488-Alexa638 tagged streptavidin with densities varying from ~300 to ~100 molecules per µm^2. The bilayers were imaged with TIRF and widefield (vertical laser). The surface densities were chosen to exceed intensity values commonly measured under experimental conditions using the same dual-tagged streptavidin stock. Ratios in TIRF and widefield were constant across all densities measured. Error bars represent standard deviation.

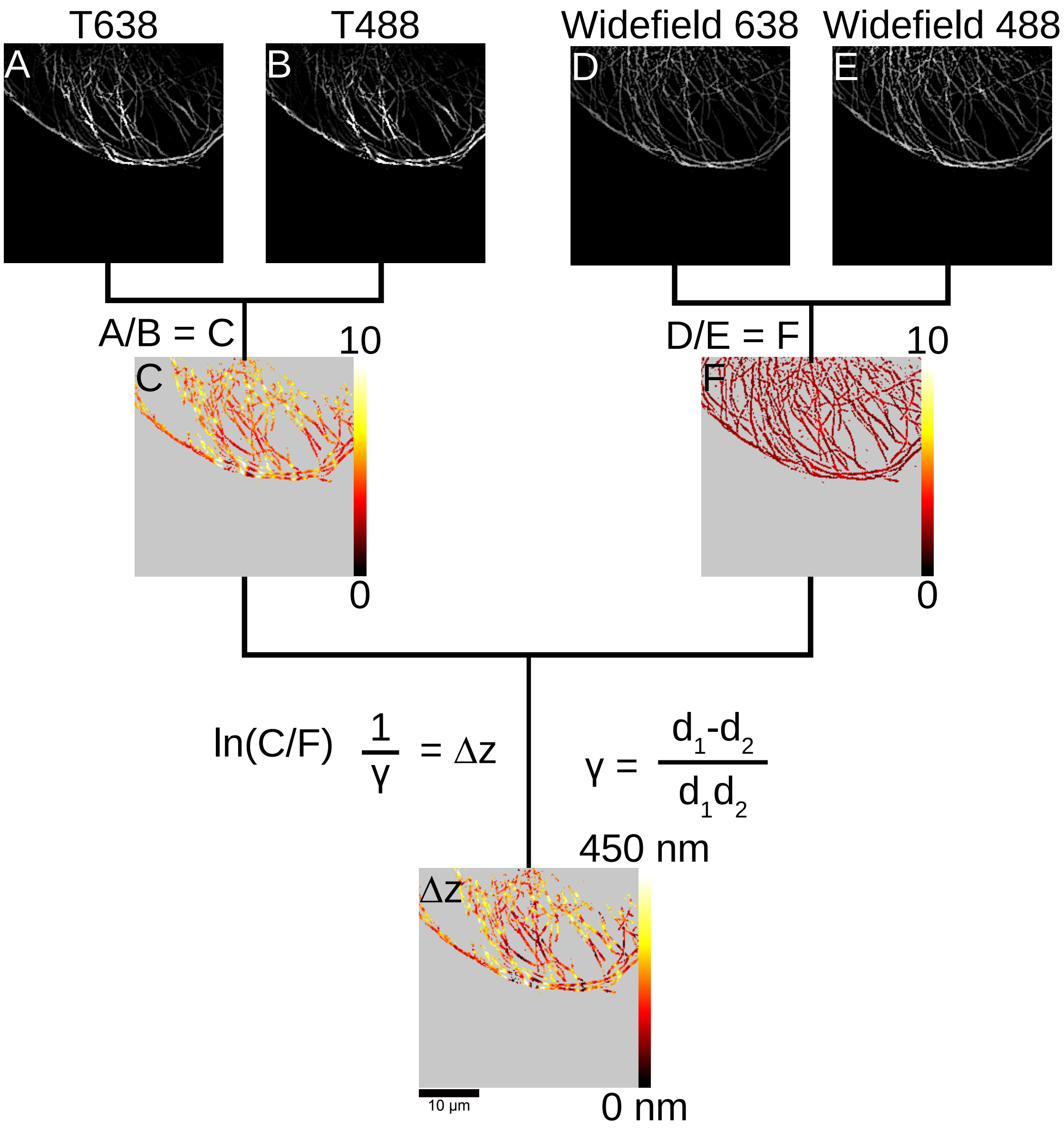

Supplementary Figure 5: Flowchart of STAR image analysis. Scheme showing how *z* is calculated from a static image with STAR. After background subtraction and flat field normalization I_{638} / I_{488} (C and F) is determined for TIRF (A and B) and widefield images (D and E). The TIRF ratio image is then divided by the widefield ratio image (reference image). Next the STAR equations are applied a to generate the Δ*z* map.

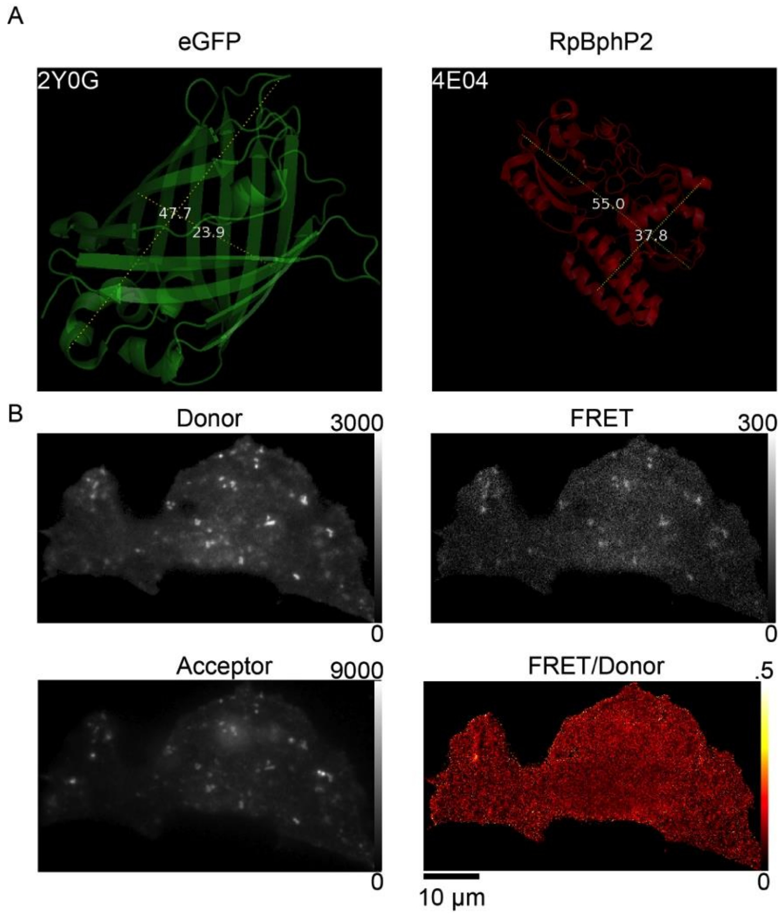

Supplementary Figure 6: FRET is uniform with EGFR-EFFP-IRFP. (A) The crystal structures and dimensions of eGFP and RpBphP2 (the bacterial phytochrome iRFP is based on). (B) Cells transfected with EGFR-EGFP-IRFP were imaged with TIRF excitation. The following images were collected: donor (488 excitation and 500/25 emission) acceptor (638 excitation and 730/55 emission) and FRET (488 excitation and 730/55 emission). The ratio of the FRET divided by donor is constant across the image. This demonstrates that there is no intermolecular FRET.

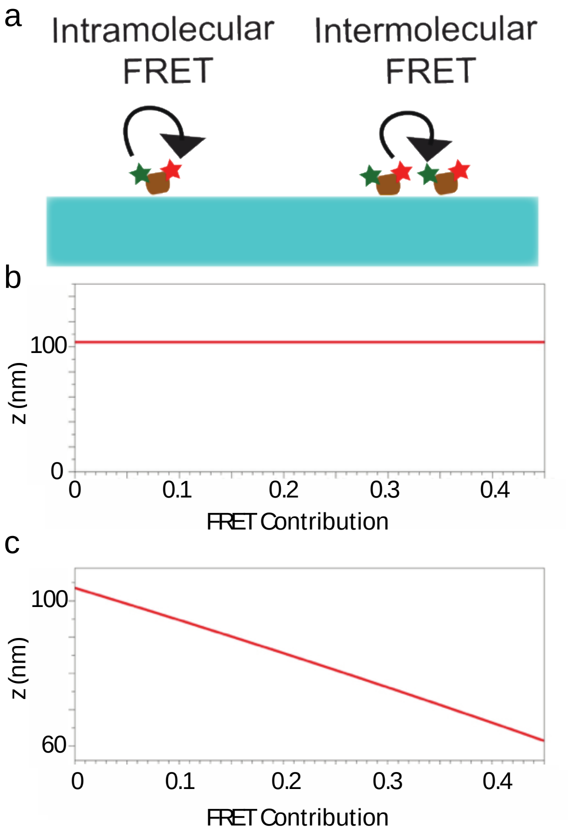

Supplementary Figure 7: a) Schematic illustrating intramolecular and intermolecular FRET. **b)** Simulation showing resulting Δz from sequential excitation of donor and acceptor over a range of intramolecular FRET efficiencies. Measured Δz is not affected by intramolecular FRET with sequential excitation. **c)** Simulation showing resulting Δz from simultaneous excitation of donor and acceptor over a range of intramolecular FRET efficiencies. Parameters for simulations in b and c were: λ = 638 and 488, θ = 72°, n_1 = 1.515, n_2 = 1.33, and true Δz = 103.5 nm.

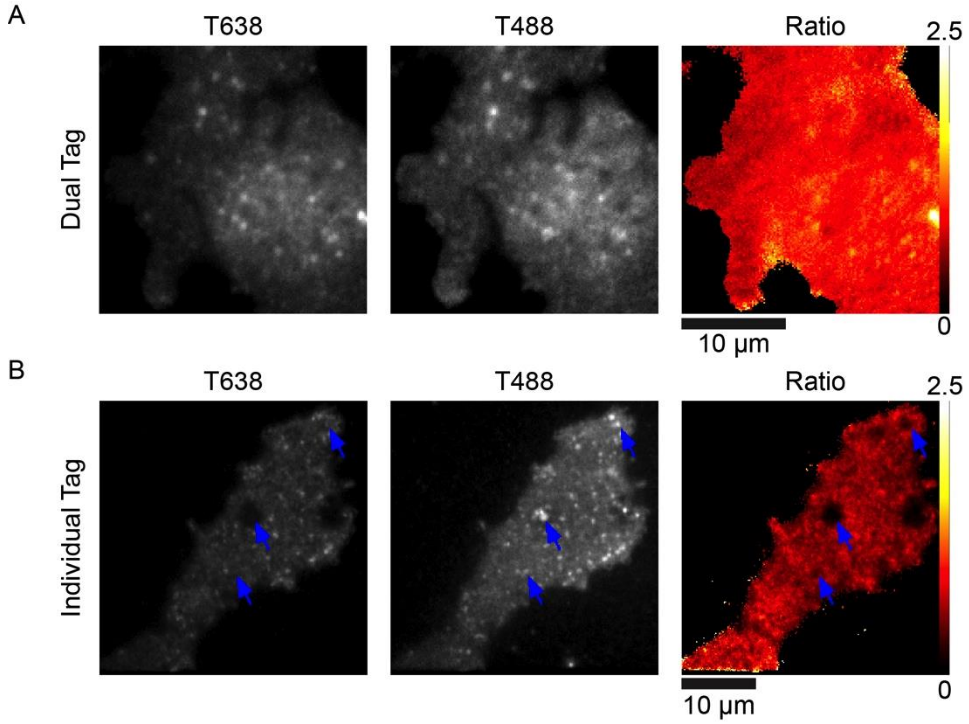

Supplementary Figure 8: Both fluorescent labels must be present on the target for STAR to function. (A) Representative images of cells transfected with EGFR-eGFP-iRFP. (B) Representative images of a cell expressing both EGFR-eGFP and EGFR-iRFP on separate constructs. Areas of disparate protein expression are apparent between the channels (blue arrows), which results in inaccurate height measurements.

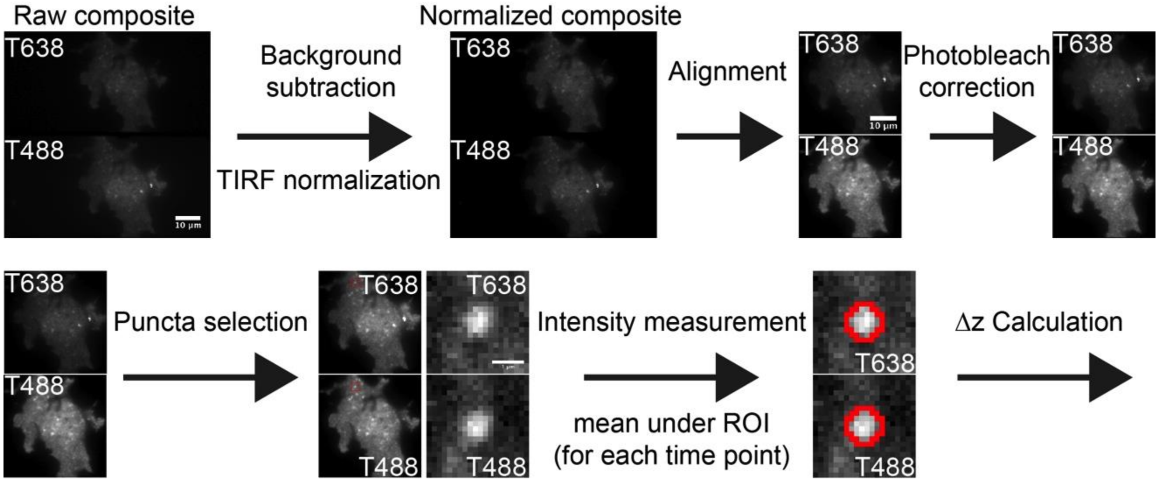

Supplementary Figure 9: Determination of *z* position from raw T638 and T488 images.

First, the raw acquired images are background subtracted based on values measured in off-cell areas. Background subtracted images are then corrected for TIRF illumination non-homogeneity via division by standard images (generated by imaging samples containing a mixture of Alexa647 and Alexa488 in solution). The normalized images are then aligned and cropped using the CAIRN Optosplit ImageJ plugin to assure that all pixels in the each channel are aligned in the respective individual images. Next, photobleaching is corrected using the EMBLtools ImageJ plugin in double exponential mode. Following this, diffraction limited puncta are selected and manually tracked over time. The MeasureStacks ImageJ plugin is then applied to these tracks to measure the intensity of the puncta in T488 and T638 for each frame. These values are then divided to produce ratios at each point. Finally, Δz is calculated from the intensity ratios as described in the methods by using either a static reference (an average of the initial time points before puncta formation) or a dynamic reference (an adjacent membrane region at each time point).

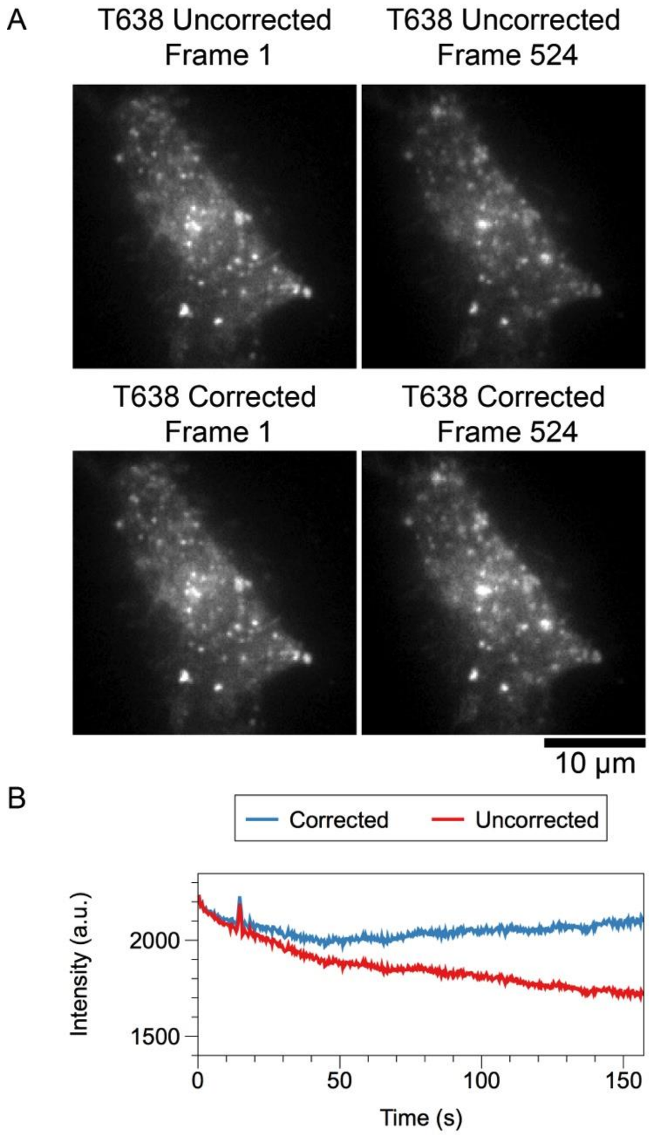

Supplementary Figure 10: Photobleaching correction. (A) Representative images of a cell transfected with EGFR-EGFP-iRFP from the first and last frames of a time lapse in the T638 channel. Images are uncorrected (top row) or corrected (bottom row) for photobleaching. All images are scaled identically. The double exponential photobleaching correction was performed using the EMBLtools ImageJ plugin. (B) The average T638 fluorescence intensity as a function of time uncorrected (red) and corrected (blue) for photobleaching.

Supplementary Note 1: Description of bead analysis software

Custom software was written in MATLAB to expedite the analysis of the silica microspheres. The following is an overview of the algorithm.

First, the constant γ is calculated from the incidence angle, excitation wavelengths, and indices of refraction. A master image stack containing the four channels, TIRF 638, TIRF 488, widefield 638, and widefield 488, for each individual field of view is created.

The user is then prompted to manually select beads from the widefield images, allowing a visual pre-screening for beads that do not have obvious defects. The user then selects background areas between beads. The average background is calculated and subtracted from each image. TIRF images are then corrected for illumination non-homogeneity using a standard fluorescent supported lipid bilayer normalization image. The diameter of each bead is calculated from the average length between peaks of a horizontal and vertical line scan though the bead. The centroid is determined by a 2D Gaussian fit to the TIRF 488 image. Next, the pixels are binned based on their distance from the bead center, and the mean intensity and coefficient of variation are calculated for each bin. Beads are then screened for uniform shape and labeling: beads with a coefficient of variation larger than 0.4 in any bin are discarded from the analysis (Supplementary Fig. 3).

For all remaining beads Δz is calculated using the centermost pixel as $z = 0$, as previously described. Theoretical values for Δz are generated for beads of various diameters using basic trigonometry to describe the profile of the bead. Finally, beads are binned together and averaged according to their diameter. The average experimental Δz is compared to the theory for beads of a given diameter.

Supplementary Note 2: The Effect of FRET on STAR Measurements

STAR is a ratiometric technique and relies on imaging two spectrally distinct fluorophores. Therefore, it is important to consider the possible effects of FRET. We considered two types of FRET: intramolecular FRET between fluorophores on the same molecule and intermolecular FRET between fluorophores on separate molecules (Supplementary Fig. 7a). We also considered two microscope configurations: sequential imaging with alternating excitations (used in the bead and microtubule experiments) and simultaneous imaging and excitation (used in the live cell EGFR experiments). The following sub-sections discuss each case in detail, and describe what corrections (if any) must be made to obtain accurate height information.

Intramolecular FRET with sequential excitation

The sample is imaged using a quad-band TIRF filter cube that passes the emission and excitation light from both channels, but excited sequentially with the wavelength corresponding to either the donor or the acceptor.

In the following treatment, it is assumed that the intramolecular FRET efficiency is constant. When the donor is excited, the collected fluorescence contains both donor and FRET emission (the quad-band cube passes the emission of both channels). Importantly, since this composite signal is solely the result of donor excitation, it follows the evanescent field decay function of the donor excitation wavelength. Direct acceptor excitation only yields acceptor emission. Therefore, intramolecular FRET does not affect the STAR measurement (Supplemental Fig. 7b). Thus, in cases of sequential donor and acceptor excitation, no correction is necessary for intramolecular FRET. The donor intensity in this case is described by the equation:

$$I_D = I_{0D}(e^{-z/d_D}) + E_{FRET}(I_{0D}(e^{-z/d_D})) \qquad \text{(eq. S1)}$$

where I_D is the total measured signal in the donor channel, I_{0D} is the donor intensity at $z = 0$, d_D is the evanescent field decay function for the donor excitation wavelength, and E_{FRET} is the FRET efficiency.

Intramolecular FRET with simultaneous excitation

When intramolecular FRET is present and the sample is imaged with simultaneous dual-channel excitation using an emission splitter, there are additional factors to consider. Importantly, all emission light from the acceptor, directly excited and excited by FRET, is collected in the acceptor channel. This means the acceptor fluorescence has components corresponding to evanescent fields of both the donor and acceptor wavelengths. This convolution of two decay functions leads to inaccurate calculation of z with STAR (Supplemental Fig. 7c). The equation for the acceptor signal is as follows:

$$I_A = I_{0A}(e^{-z/d_D}) + E_{FRET}(I_{0A}(e^{-z/d_A})) \quad \text{(eq. S2)}$$

where I_A is the total measured signal in the acceptor channel, I_{0A} is the acceptor intensity at z = 0, and d_A is the evanescent field decay function for the acceptor excitation wavelength.

Data can be corrected for intramolecular FRET by subtracting the FRET contribution from the acceptor image using standard techniques. The FRET contribution can be measured by exciting with only the donor excitation wavelength.

Intermolecular FRET sequential or simultaneous excitation

Intermolecular FRET is not tolerated when running dynamic STAR imaging. In the case of sequential excitation, the FRET signal will contribute to the donor channel as discussed above. In a fixed sample, any intermolecular FRET can be corrected by using the widefield ratio image to define z = 0. This pixel-by-pixel correction includes any variations in local FRET efficiency. In the case of simultaneous excitation, the only way to correct the discrepancy is to measure FRET. As this would greatly slow down the rate of imaging for dynamic cell experiments, it is necessary to design constructs with minimal intermolecular FRET.

Supplementary Note 3: STAR bias in measurements of vesicle height

STAR measurements of co-localized objects with different z positions introduces a small bias based on the geometry. Due to the fact that the evanescent fields are more intense near the coverslip, STAR signal is weighted towards objects lower in z. In order to estimate this effect in EGFR endocytosis, a vesicle was modeled by a sphere (200 nm diameter). Assuming uniform coverage of dual tagged EGFR, the overall fluorescence was numerically calculated. Using this calculation, the height determined by STAR was 71 nm; a 29 nm deviation from the geometric center, 100 nm. It should be noted that the deviation depends on the distribution of the fluorophores and the geometry of the object being measured, as well as the absolute z position.
